# Standard Versus Advanced Protective Measures in a COVID-Free Surgical Pathway

**DOI:** 10.7759/cureus.31227

**Published:** 2022-11-08

**Authors:** Fabio Frosio, Riccardo Masserano, Fabio Colli, Luca Portigliotti, Fabio Maroso, Filadelfio Massimiliano Nicolosi, Oscar Soresini, Raffaele Romito

**Affiliations:** 1 Department of General Surgery, Maggiore Hospital, Novara, ITA

**Keywords:** covid-19 retro, inpatient ward management, protective measures, general surgery, covid-free surgical pathway, covid-19

## Abstract

Introduction

The importance of coronavirus disease (COVID)-free surgical pathways during the coronavirus disease 2019 (COVID-19) pandemic has been demonstrated. However, the extent of protective measures to be applied against severe acute respiratory syndrome coronavirus 2 (SARS-CoV-2), particularly before vaccines became available, remained unclear.

Methods

This retrospective study included all SARS-CoV-2-negative patients admitted to the COVID-free pathway of a regional abdominal surgery hub center in Northern Italy over 12 months, before the vaccination campaign. During the first seven months, basic protective measures against SARS-CoV-2 were adopted (surgical masks, swabs for symptomatic patients, and intra- or interhospital transfers), since patients were treated as effectively negative (standard management). During the last five months, advanced measures were implemented (enhanced personal protections and systematic control swabs), as patients were considered potentially positive (advanced management). The aim of this article was to compare SARS-CoV-2 incidence and surgical outcomes in these periods.

Results

A total of 283 and 194 patients were admitted under standard and advanced management, respectively; pre-admission data differed only in the rate of previous SARS-CoV-2 infection (2.5% versus 6.7%, p= 0.034). The SARS-CoV-2 incidence was 3.9% and 3.1% for standard and advanced periods, respectively (p = 0.835). Two internal outbreaks developed during the standard phase. The advanced protocol significantly increased the rate of patients re-tested for SARS-CoV-2 (83% versus 41.7%, p < 0.001) and allowed early detection of all infections, which remained sporadic. Surgical outcomes were similar.

Conclusions

Advanced management was instrumental in detecting positive patients early and preventing outbreaks, without affecting surgical results; accordingly, it stands as a reproducible model for future pandemic scenarios.

## Introduction

Northern Italy was the first European area to be hit by coronavirus disease 2019 (COVID-19), in late February 2020. On March 9th, the day the Italian government announced a national lockdown, there were nearly 6,000 confirmed cases, with a daily increase of about 25% [1]. Italian hospitals were faced with the huge challenge of admitting more and more patients with COVID-19, especially in intensive care units (ICU). For this reason, a major reorganization of hospitals was carried out. Surgical departments were particularly affected. Emergency surgery was clearly guaranteed, while elective activity was restricted to oncological cases only [2].

In order to ensure these procedures, our hospital, located in Northern Italy and close to the epicenter of the pandemic, quickly established a COVID-free surgical pathway, in which operating theaters, inpatient wards, and ICU were not shared with COVID-19 patients. To enter this pathway in both elective and emergency settings, severe acute respiratory syndrome coronavirus 2 (SARS-CoV-2) was tested using real-time reverse transcriptase polymerase chain reaction (rRT-PCR) on nasopharyngeal swabs [3]. The surgical pathway actually maintained a COVID-free status with standard protective measures for seven months, until two internal COVID-19 outbreaks prompted a major change in its management. Since then, more stringent measures to prevent infection were adopted, as every patient was managed as a potential positive.

The importance of creating COVID-free surgical pathways during the pandemic is well known; on the other hand, the extent of protective measures applied against SARS-CoV-2 in these pathways, especially before the vaccination campaign, is an issue that remains unclear in the literature. The aim of this article was to assess SARS-CoV-2 infection within our COVID-free surgical pathway after one year of the pandemic, in the time frame between its onset and the beginning of the large-scale vaccination campaign. In particular, the seven-month period of standard measures and the five-month period of advanced measures were compared to investigate which of the two systems provided a higher level of protection against the virus. Surgical results were also analyzed to determine whether the two systems may have influenced surgical practice.

## Materials and methods

Study population and pre-admission data

This retrospective study included all patients who entered the COVID-free surgical pathway, in both elective and emergency settings, at the Department of General Surgery of the Maggiore Hospital in Novara, Italy, from March 15th, 2020, to March 14th, 2021. None of them had previously received any dose of the anti-COVID vaccine. To be admitted to the COVID-free surgical pathway, all patients had to test negative for SARS-CoV-2 on rRT-PCR on nasopharyngeal swabs. On the other hand, patients who tested positive did not enter the COVID-free surgical pathway and were not considered. Patients with a known previous SARS-CoV-2 infection who still tested positive were not considered either. Data were prospectively collected.

The following pre-admission data were considered: patient’s age and sex, diagnosis, elective or emergency admission status, comorbidities, and previous SARS-CoV-2 infection.

This study was approved by the institutional review board and conducted in accordance with the Declaration of Helsinki.

COVID-19 testing for surgical admission

Patients scheduled for elective surgery were given an appointment for COVID-19 testing at least 72 or 48 hours before the procedure, at the dedicated facility just outside the hospital. After the off-site nasopharyngeal swab for rRT-PCR, each patient returned home and observed a self-quarantine until admission. If the test was positive, surgery was postponed. If the test was negative and no COVID-19 symptoms occurred in the meantime, the patient could enter the COVID-free pathway and undergo surgery.

On the other hand, patients requiring emergent surgical care were tested for SARS-CoV-2 infection directly in the emergency department. If both nasopharyngeal swab and chest imaging were negative, patients could enter the COVID-free pathway; if the nasopharyngeal swab was positive, patients were moved either to the COVID-19 operating theater or to the COVID-19 inpatient unit. Patients with a known previous SARS-CoV-2 infection who still tested positive did not enter these pathways but a dedicated zone with single rooms, where healthcare providers had to wear enhanced personal protective equipment (PPE). In case of life-threatening conditions, the result of the swab was not awaited, and patients were managed as a potential positive; until SARS-CoV-2 status was clarified, they did not enter the COVID-free pathway, and healthcare providers used PPE.

Intraoperative bronchoalveolar lavage (BAL) for rRT-PCR was performed for all patients potentially requiring postoperative ICU admission, to determine whether they should be admitted to COVID or COVID-free ICU. Moreover, in the context of emergency surgery, it was also used to confirm a patient’s status when in doubt (e.g., a negative nasal swab in a patient with mild symptoms or unclear radiological findings).

Standard management

During the seven-month period of standard management, patients could share double rooms, with beds at least two meters apart and a curtain in between. Patients had to wear surgical face masks. The same protective measure had to be undertaken by healthcare providers; they also had to use disposable gloves and perform accurate hand disinfection before and after approaching each patient. Nasopharyngeal swabs were not repeated systematically but only for patients who presented COVID-19 symptoms, for patients undergoing further procedures (surgical revisions, interventional radiology, or endoscopy), and for patients transferred to other departments, hospitals, or nursing homes. Healthcare providers were regularly tested every month. If a patient tested positive during the hospital stay, he or she was transferred to the COVID ward, and the room was disinfected; all healthcare providers and patients in the same section of the ward were tested twice, five to seven days apart. Visitors were not admitted.

Advanced management

With the transition to advanced management, while the admission criteria remained unchanged, more restrictive protective measures were applied. Firstly, healthcare providers had to use enhanced PPE: disposable surgical caps, N95 masks, inner disposable latex gloves, eye coverings (full face visors or goggles), isolation gowns, and outer disposable latex gloves [4]. PPE was available on a small table outside each room, with the proper disposal containers. Secondly, control nasopharyngeal swabs were repeated weekly, both for patients and healthcare providers; moreover, with the exception of those scheduled for day-case surgery and those staying only one night, all patients were systematically tested with an additional swab the day before discharge, regardless of their destination. Visitors were still not authorized. The protocol in case of a positive case did not change either.

Hospitalization data and follow-up

The following hospitalization data were considered: surgical or medical treatment, complexity of surgery, general or locoregional anesthesia, rRT-PCR result on the eventual intraoperative BAL, ICU admission, rRT-PCR result on nasopharyngeal swabs during the hospital stay, length of hospital stay, surgical and pulmonary complications, detection of SARS-CoV-2 infection, and 90-day mortality. An internal outbreak of SARS-CoV-2 was defined as the simultaneous presence of three or more positive patients in the inpatient ward. Complexity of surgery was ranked as minor, intermediate, or major, according to the Clinical Coding & Schedule Development Group [5]. Surgical complications were classified using the Clavien-Dindo classification [6]. Pulmonary complications included lobar, lobular, or interstitial pneumonia [7], acute pulmonary embolism (PE), acute respiratory distress syndrome (ARDS), and/or unexpected need for ventilatory support in the absence of these conditions. Patients were followed up at least until day 90 after discharge.

Statistical analysis

Demographic, pre-admission, and hospitalization data were analyzed, as well as postoperative outcomes and detection of SARS-CoV-2 infection. Patients admitted under standard and advanced management were compared. Quantitative variables were expressed as median (with interquartile range (IQR)) and compared using Student’s t-test, while qualitative variables were expressed as percentages and compared using the chi-squared test or Fisher’s exact test when appropriate. All statistical analyses were performed using Prism 6.0 (GraphPad Software, La Jolla, CA, USA); statistical significance was attested for p values ≤ 0.05.

## Results

Studied population and pre-admission data

A total of 477 patients were included. Among them, there were 252 (52.8%) males and 225 (47.2%) females, with a median age of 68 years (IQR: 56-78).

There were 192 (40.3%) patients admitted for elective surgical procedures, while there were 285 (59.7%) patients admitted from the emergency department, of whom 209 (73.3%) underwent surgical treatment. Compared to the previous 12 months, elective and emergency cases actually decreased by 39.8% and 18.9%, respectively. Overall, there were 198 (41.5%) cancer patients. With regard to the main comorbidities, 76 patients (15.9%) had ischemic or arrhythmic heart disease, 62 (13%) had diabetes mellitus, 36 (7.5%) had chronic obstructive pulmonary disease (COPD), and 55 (11.5%) were obese (body mass index (BMI) ≥ 30 kg/m^2^). A total of 20 (4.1%) patients had a known history of SARS-CoV-2 infection.

Standard versus advanced management

Overall, 283 patients were admitted during the seven-month standard management phase, whereas 194 patients were admitted during the five-month advanced protocol. The two groups are compared in Table 1.

**Table 1:** Standard versus advanced management of COVID-free surgical pathway COVID-19, coronavirus disease 2019; SARS-CoV-2, severe acute respiratory syndrome coronavirus 2; ASA score, American Society of Anesthesiologists score; rRT-PCR, real-time reverse transcriptase polymerase chain reaction; BAL, bronchoalveolar lavage; ICU, intensive care unit; ARDS, acute respiratory distress syndrome; PE, acute pulmonary embolism; IQR: interquartile range

	Standard management, n = 283	Advanced management, n = 194	p
Demographic data			
Age, years, median (IQR)	69 (57-78)	68 (55-78)	0.801
Males/females, n (%)	153 (54.1%)/130 (45.9%)	99 (51%)/95 (49%)	0.575
Pre-admission data			
Elective/Emergency hospital admission, n (%)	119 (42%)/164 (58%)	73 (37.6%)/121 (62.4%)	0.383
Previous SARS-CoV-2 infection, n (%)	7 (2.5%)	13 (6.7%)	0.034
Cancer/non-cancer patients, n (%)	124 (43.8%)/159 (56.2%)	74 (38.1%)/120 (61.9%)	0.254
Comorbidities			
Ischemic or arrhythmic heart disease, n (%)	49 (17.3%)	27 (13.9%)	0.385
Diabetes mellitus, n (%)	41 (14.5%)	21 (10.8%)	0.303
Chronic obstructive pulmonary disease, n (%)	19 (6.7%)	17 (8.8%)	0.512
Obesity, n (%)	31 (11%)	24 (12.3%)	0.741
Surgical treatment			
Operated patients, n (%)	242 (85.5%)	159 (81.9%)	0.361
Major surgery, n (%)	53 (21.9%)	34 (21.4%)	0.902
Intermediate or minor surgery, n (%)	189 (78.1%)	125 (78.6%)	0.902
Laparoscopic surgery, n (%)	76 (31.4%)	53 (33.3%)	0.768
Robotic surgery, n (%)	23 (9.6%)	19 (11.9%)	0.538
General anesthesia, n (%)	223 (92.1%)	143 (89.9%)	0.557
ASA score 1-2, n (%)	147 (60.7%)	110 (69.2%)	0.106
ASA score 3-5, n (%)	95 (39.3%)	49 (30.8%)	0.106
rRT-PCR on intraoperative BAL, n (%)	78 (32.2%)	52 (32.7%)	0.921
Patients tested positive on BAL, n (%)	1 (1.3%)	0 (0%)	1.000
Postoperative ICU admission, n (%)	25 (10.3%)	14 (8.8%)	0.731
Surgical complications	57 (23.6%)	36 (22.6%)	0.927
Clavien-Dindo I, n (%)	6 (10.5%)	2 (5.6%)	0.478
Clavien-Dindo II, n (%)	13 (22.8%)	9 (25%)	0.807
Clavien-Dindo IIIa, n (%)	12 (21.1%)	8 (22.2%)	1.000
Clavien-Dindo IIIb, n (%)	14 (24.6%)	8 (22.2%)	0.993
Clavien-Dindo IVa, n (%)	2 (3.5%)	2 (5.5%)	0.635
Clavien-Dindo IVb, n (%)	0 (0%)	1 (2.8%)	0.816
Clavien-Dindo V, n (%)	10 (17.5%)	6 (16.7%)	0.913
Pulmonary complications			
Lobar or lobular pneumonia	12 (4.2%)	6 (3.1%)	0.626
Interstitial pneumonia	6 (2.1%)	3 (1.5%)	0.744
ARDS	2 (0.7%)	1 (0.5%)	1.000
PE	2 (0.7%)	2 (1%)	1.000
Need for ventilatory support without other conditions	6 (2.1%)	4 (2.1%)	1.000
COVID-19 testing			
Patients re-tested with rRT-PCR on nasopharyngeal swabs during hospital stay, n (%)	118 (41.7%)	161 (83%)	<0.001
Main reason for re-testing patients			
Symptoms suggestive of COVID-19, n (%)	22 (18.6%)	11 (6.8%)	0.005
Need for further procedures, n (%)	19 (16.1%)	12 (7.5%)	0.038
Transfers, n (%)	24 (20.4%)	12 (7.5%)	0.003
Positive cases in the ward, n (%)	53 (44.9%)	0 (0%)	<0.001
Advanced management protocol, n (%)	-	126 (78.2%)	<0.001
SARS-CoV-2 infection			
Patients tested positive on nasopharyngeal swab during hospital stay, n (%)	10 (3.5%)	6 (3.1%)	0.997
Overall SARS-CoV-2 incidence	11 (3.9%)	6 (3.1%)	0.835
COVID-19 outbreaks in the ward, n	2	-	-
Mortality due to SARS-CoV-2, n (%)	1 (0.4%)	0 (0%)	1.000
Length of hospital stay, days, median (IQR)	7 (4-10)	6 (4-8)	0.215

The two groups were absolutely homogeneous in terms of demographic data. Regarding pre-admission data, there were no statistically significant differences in elective or emergency admission status, the latter being predominant; the number of patients with cancer and major comorbidities did not differ either. On the other hand, the rate of previous SARS-CoV-2 infection was significantly higher for patients admitted during the advanced protocol period (6.7% versus 2.5%, p = 0.034).

Only a minority of patients, all admitted from the emergency department, received medical treatment exclusively or combined with interventional radiology or endoscopy: 41/283 (14.5%) and 35/194 (18%) for the standard and advanced period, respectively.

Most patients actually underwent surgery: 85.5% and 81.9% of the above periods, respectively. In both periods, major surgery (essentially hepatic, pancreatic, and rectal resections) accounted for approximately 22% of overall surgical procedures, while intermediate or minor surgery (including almost all emergency surgeries as well) for about 78%; day-case surgery was nearly inexistent throughout the whole year. The two groups were homogeneous also with respect to the surgical approach, as laparoscopic and robotic procedures were performed in similar percentages under standard and advanced management (31.4% and 9.6% versus 33.3% and 11.9% of all surgical procedures). There were no differences between them from an anesthesiologic point of view either, taking into account the American Society of Anesthesiologists (ASA) score of the patients and the type of anesthesia. Intraoperative BAL for rRT-PCR was also performed in identical amounts: 32.2% for standard management patients and 32.7% for advanced management patients. Only one patient among the former (1.3%) tested positive for SARS-CoV-2. This patient was a 78-year-old female undergoing the Whipple procedure, who died on postoperative day 10 due to severe COVID-19 interstitial pneumonia, in the absence of abdominal complications. Postoperative ICU admission concerned 10.3% of patients in the standard period versus 8.8% of patients in the advanced period (p = 0.731). A total of 57/242 (23.6%) and 36/159 (22.6%) patients experienced surgical complications in the two periods, respectively (p = 0.927), with a similar pattern according to the Clavien-Dindo grading system.

Considering all patients, similar rates of pulmonary complications were reported in the two groups, lobar or lobular pneumonia being the most frequent. Ventilatory support in the absence of an evident respiratory disease was needed in identical proportions.

With the establishment of the advanced management, the rate of patients re-tested for SARS-CoV-2 during hospital stay significantly increased at 161/194 (83%), compared to 118/283 (41.7%) of the standard management (p < 0.001). Length of hospital stay became slightly shorter than previously, six (IQR: 4-8) days versus seven (IQR: 4-10) days, although not statistically significant (p = 0.215). Importantly, the vast majority of advanced management patients (126/194, 78.2%) were re-tested only as a result of the new protocol. On the other side, reasons for re-testing during the standard management period were as follows: surveillance after the detection of a positive case (53/118, 44.9%), patient transfers (24/118, 20.4%), symptoms suggestive of COVID-19 (22/118, 18.6%), and need for further procedures (19/118, 16.1%).

There were 10/283 (3.5%) patients under standard and 6/194 (3.1%) patients under advanced management who tested positive on nasopharyngeal swabs during hospital stay; all of the latter were asymptomatic, and their swabs were taken exclusively because of the advanced protocol. None of the surgical patients (8/10 and 4/6, respectively) had actually undergone intraoperative BAL.

Taking into account the abovementioned patient found positive on BAL, the overall SARS-CoV-2 infection incidence within the COVID-free surgical pathway was 3.9% (11/283) and 3.1% (6/194) for the standard and advanced period, respectively. In particular, during the first period, two distinct outbreaks occurred, with three and four patients testing positive at the same time; two healthcare providers, one of whom was symptomatic with fever and anosmia, also tested positive during the second outbreak. On the opposite, in the second period, only isolated infections were detected. Overall, 3/283 (1.1%) patients developed viral interstitial pneumonia versus 0/194 (0%), and mortality for COVID-19 was 1/283 (0.4%) versus 0/194 (0%) for standard and advanced periods, respectively.

## Discussion

Surgical activity has been heavily impacted by the COVID-19 pandemic worldwide. In particular, non-oncological elective procedures were postponed almost everywhere, in order to increase the intensive care capacity of hospitals. Of course, in this scenario, the key principle for safely providing both urgent and oncological surgery was the establishment of a COVID-free patient flow process. The importance of these dedicated pathways was demonstrated early on: in August 2020, an international multicenter study actually reported that pulmonary complications, SARS-CoV-2 infection, and mortality were significantly less prevalent for patients undergoing oncological surgery within a COVID-free pathway; specifically, this was conceived as a well-defined setting in which operating room, critical care, and inpatient ward were completely separated from COVID-19 patients [8].

The detection of viral RNA by rRT-PCR has been recognized as the gold standard diagnostic test for SARS-CoV-2 infection, and rRT-PCR on nasopharyngeal swabs was globally used to determine the admission of patients to the COVID-free pathways [9]. However, it is well known that rRT-PCR sensitivity increases proportionally with the viral load and also depends on where the sample is taken from, being lower for nasal and oropharyngeal samples than for BAL [10]. Therefore, the risk of admitting false negatives to a COVID-free surgical pathway may be considerable, especially if patients are still in the early incubation phase or if nasopharyngeal swabs are incorrectly collected [11,12]. In addition, patients scheduled for elective surgery may be infected after the off-site swab if they do not comply with the pre-admission self-quarantine.

So far, many reports have focused on perioperative measures to be taken for COVID-19 patients [13-16], but none have really addressed the issue of the level of protection to be adopted in a surgical pathway where, although patients are supposed to be negative, there may be possible false negatives.

In March 2020, at the time when Lombardy was the first European region to be affected by the COVID pandemic, the Maggiore Hospital in Novara (located a few kilometers from Lombardy) set up one of the first COVID-free pathways for surgery. Given that all patients tested negative for SARS-CoV-2 infection on the pre-admission swab, a standard level of protective measures within the pathway was considered adequate to ensure the safety of both patients and healthcare providers. Several months later, when vaccines were not yet available, two internal COVID-19 outbreaks motivated the adoption of more advanced protective measures.

This provided an opportunity to compare two different management systems, consecutively applied in the same environment and by the same operators: standard management, where the patient is treated as a definite negative, and advanced management, where he or she is managed as a potential positive case. From an epidemiological point of view, the standard management period covered the first COVID wave (spring 2020), while the advanced management period included the second wave (fall-winter 2020). Both periods had similar infection rates at a regional level and involved national lockdowns [17]. The two management systems included patients who were absolutely homogeneous with regard to demographic, anamnestic, and pre-admission data. The only significant difference concerned the rate of previous SARS-CoV-2 infection, which was higher for patients admitted under advanced management: as this phase was introduced several months later, this result seems to reflect the progression of the pandemic over time. Moreover, the same proportion of patients in both groups underwent surgical treatment, either elective or emergent, which was also of comparable complexity.

In terms of surgical outcomes, there were no differences in complications and 90-day mortality between the standard and advanced phases. While during the former, the patient was regularly seen by several physicians and nurses throughout the day, the need for enhanced PPE in the latter meant that usually, only one physician could examine the patient, in the morning and in the afternoon. Access to the patient’s room by nurses was also rationalized in the latter phase. Nevertheless, as the surgical outcomes show, the quality of care was not impaired at all, which clearly proves the expertise of the whole surgical team. Probably, the awareness of having to optimize contact with the patient further increased the focus of healthcare providers. Shorter hospital stays during the advanced management can be considered another effect of the optimization effort of this phase.

The rate of SARS-CoV-2 infections in the two types of management was absolutely comparable. During the standard phase, however, two separate outbreaks occurred, involving seven patients (one of whom died) and two healthcare providers. On the other hand, during the advanced management, all infections were sporadic, as the use of enhanced PPE prevented COVID-19 outbreaks. Importantly, the six positive patients were all asymptomatic and were actually identified through the new protocol, which involved predischarge and weekly surveillance swabs for almost every patient.

Of course, the need for enhanced PPE and systematic control nasal swabs represents a very high cost in terms of time and expense, compared to a standard approach; for this reason, an advanced policy may not be feasible everywhere and at all times, despite its definite advantages. The main limitation of the present study is actually the lack of economic cost analysis, for which further studies are needed; another limitation is the retrospective approach, although data were prospectively collected.

## Conclusions

In conclusion, the present study shows that the presence of positive patients in a COVID-free surgical pathway is possible, being considered as false negatives on the pre-admission swab or being infected afterward. In this context, advanced protective measures have proved to be instrumental in detecting positive cases early and preventing internal outbreaks, without affecting surgical outcomes.

Thus, the main strength of this work is the identification of a surgical pathway management system that protected both patients and healthcare providers from the spread of SARS-CoV-2, prior to the start of the vaccination campaign. Our results may be considerably useful now, given the unknown duration of the immune protection currently provided by vaccines, especially in those countries where most of the population has not yet been vaccinated. Remarkably, this study might represent a valuable option for future pandemic scenarios, as it defines and validates a reproducible model. Based on our experience, the advanced management model should be rapidly implemented in hospital wards in case of high COVID-19 community rates and in the event of novel, severe, and vaccine-resistant SARS-CoV-2 variants. Indeed, advanced protective measures could be used to control and limit the spread of any future airborne viral diseases before vaccines are available, without impairing community care and the safety of healthcare providers.
